# Study on the Mechanism of MC5R Participating in Energy Metabolism of Goose Liver

**DOI:** 10.3390/ijms24108648

**Published:** 2023-05-12

**Authors:** Jinqi Zhang, Ya Xing, Fangbo Li, Ji’an Mu, Tongjun Liu, Jing Ge, Minmeng Zhao, Long Liu, Daoqing Gong, Tuoyu Geng

**Affiliations:** 1 College of Animal Science and Technology, Yangzhou University, Yangzhou 225009, China; zjq397495452@outlook.com (J.Z.); xingya325@163.com (Y.X.); qq971409175@outlook.com (F.L.); m18326768696@outlook.com (J.M.); c28942662@gmail.com (T.L.); gejing@yzu.edu.cn (J.G.); zhaominmeng123@163.com (M.Z.); liujiaolong688@sina.com (L.L.); 2 Joint International Research Laboratory of Agriculture and Agri-Product Safety of the Ministry of Education of China, Yangzhou University, Yangzhou 225009, China

**Keywords:** goose, MC5R, liver, differentially expressed gene, energy metabolism

## Abstract

Nutrition and energy levels have an important impact on animal growth, production performance, disease occurrence and health recovery. Previous studies indicate that melanocortin 5 receptor (MC5R) is mainly involved in the regulations of exocrine gland function, lipid metabolism and immune response in animals. However, it is not clear how MC5R participates in the nutrition and energy metabolism of animals. To address this, the widely used animal models, including the overfeeding model and the fasting/refeeding model, could provide an effective tool. In this study, the expression of *MC5R* in goose liver was first determined in these models. Goose primary hepatocytes were then treated with nutrition/energy metabolism-related factors (glucose, oleic acid and thyroxine), which is followed by determination of *MC5R* gene expression. Moreover, *MC5R* was overexpressed in goose primary hepatocytes, followed by identification of differentially expressed genes (DEGs) and pathways subjected to MC5R regulation by transcriptome analysis. At last, some of the genes potentially regulated by MC5R were also identified in the in vivo and in vitro models, and were used to predict possible regulatory networks with PPI (protein–protein interaction networks) program. The data showed that both overfeeding and refeeding inhibited the expression of *MC5R* in goose liver, while fasting induced the expression of *MC5R*. Glucose and oleic acid could induce the expression of *MC5R* in goose primary hepatocytes, whereas thyroxine could inhibit it. The overexpression of *MC5R* significantly affected the expression of 1381 genes, and the pathways enriched with the DEGs mainly include oxidative phosphorylation, focal adhesion, ECM–receptor interaction, glutathione metabolism and MAPK signaling pathway. Interestingly, some pathways are related to glycolipid metabolism, including oxidative phosphorylation, pyruvate metabolism, citrate cycle, etc. Using the in vivo and in vitro models, it was demonstrated that the expression of some DEGs, including *ACSL1*, *PSPH*, *HMGCS1*, *CPT1A*, *PACSIN2*, *IGFBP3*, *NMRK1*, *GYS2*, *ECI2*, *NDRG1*, *CDK9*, *FBXO25*, *SLC25A25*, *USP25* and *AHCY*, was associated with the expression of *MC5R*, suggesting these genes may mediate the biological role of MC5R in these models. In addition, PPI analysis suggests that the selected downstream genes, including *GYS2*, *ECI2*, *PSPH*, *CPT1A*, *ACSL1*, *HMGCS1*, *USP25* and *NDRG1*, participate in the protein–protein interaction network regulated by *MC5R*. In conclusion, MC5R may mediate the biological effects caused by changes in nutrition and energy levels in goose hepatocytes through multiple pathways, including glycolipid-metabolism-related pathways.

## 1. Introduction

The liver is an important organ for nutrition and energy metabolism in animals [1]. When nutrition and energy are excessive, glucose is not only converted into glycogen and stored in the liver, but also converted into fat. The latter is then transported via blood to adipose tissue for storage. In contrast, when nutrition and energy cannot meet the needs for normal physiological function and production, the liver will break down glycogen into glucose or increase gluconeogenesis to maintain blood glucose levels [2,3,4]. Therefore, the liver plays a pivotal role in nutrition and energy metabolism.

Appropriate nutrition and energy levels are necessary for animals to maintain various physiological functions. Changes in nutrition and energy levels may cause a large effect on animals. For example, in the rearing process of Peking ducks, raising the levels of metabolizable energy and crude protein can significantly increase the body weight of ducks, whereas feed intake and the ratio of feed to weight are decreased [5,6]. It is also reported that increasing protein and energy levels can improve chicken antibody titers to pathogens [7]. However, excessive intake of an energy-rich diet can lead to some metabolic diseases. For example, some studies show that high-energy and low-protein diets can induce excessive abdominal fat, an obvious reduction in egg production and the occurrence of fatty liver hemorrhagic syndrome in laying hens [8,9]. In conclusion, changes in nutrition and energy levels have a great influence on the growth and development, disease occurrence, and production performance of animals.

The overfeeding model, the fasting/refeeding model and the high sugar/fat diet model are widely used in nutrition or energy metabolism-related studies in animals. Using these models, a number of studies have revealed that many genes and pathways are influenced by changes in nutrition and energy levels. For example, using the fasting/refeeding model, Xu et al. [10] demonstrated that fasting decreased the expression of fat synthesis genes (Stearoyl-CoA Desaturase (*SCD*), glucocorticoid receptor (*GR*) and fatty acid synthase (*FAS*)) in the liver and increased the expression of lipolytic genes (AMP-activated protein kinase alpha subunit (*AMPKα*), *AMPKβ* and *AMPKγ*) in the hypothalamus. Liver transcriptome analysis indicates that fasting affects the nutrition metabolism in goose liver, especially the signaling pathways related to lipid metabolism. Refeeding not only affects lipid metabolism pathways, but also affects glucose and amino acid metabolism pathways. Among these pathways, the peroxisome proliferator-activated receptor (PPAR) signaling pathway may play an important role in lipid metabolism [11]. Furthermore, using the overfeeding study model, liver transcriptome analysis indicates that liver transcriptome is characterized by metabolic pathways at the early stage of overfeeding, while the cell growth and death pathway and the immune diseases pathway are also significantly enriched with differentially expressed genes at the later stage of overfeeding [12]. Overall, using these models may help to reveal the molecular mechanisms by which the changes in nutrition and energy metabolism affect animal physiology and health, and thus provide a theoretical reference for animal production.

Studies have shown that changes in the levels of nutrition and energy metabolism can affect the expression of the genes involved in the melanocortin/melanocortin receptor (MC/MCR) system. For example, fasting can induce mRNA expression of Agouti-related protein (*AgRP*), melanocortin 4 receptor (*MC4R*) and neuropeptide Y (*NPY*) genes in the hypothalamus of quail and chicken [13,14]. This suggests that the MC/MCR system may be involved in the regulation of nutrition and energy metabolism in animals. Indeed, melanocortins (including adrenocorticotropic hormone (ACTH), alpha, beta, gamma-melanocyte-stimulating hormone (α, β, γ-MSH), etc.) are known to play an important role in food intake, blood pressure regulation, hormone secretion regulation, blood glucose regulation and energy expenditure [15,16]. Moreover, the expression of melanocortin receptors, including melanocortin 1 receptor (MC1R), melanocortin 2 receptor (MC2R), melanocortin 3 receptor (MC3R), MC4R and melanocortin 5 receptor (MC5R), in different tissues is related to cell type, and these receptors play a key role in mediating the biological effects of melanocortins [17,18,19]. Different receptors have different affinities for a variety of melanocortins and have different biological functions. For example, MC1R is mainly involved in inflammatory response and pigmentation, and also plays an important role in skin tone control and in the repair of ultra violet (UV) induced DNA damage [20]. MC2R mainly participates in the synthesis of a variety of steroids. MC3R is associated with feeding behavior and metabolic homeostasis. MC4R is involved in the formation of satiety, and point mutations in MC4R can induce obesity in animals [21].

Compared to other MCRs, there are relatively few studies on MC5R. It is known that MC5R has a broad expression profile, it is not only expressed in the central nervous system, but also in the skin, skeletal muscle, fat, lymph nodes, duodenum, liver, kidney, testis, ovary, uterus and many exocrine glands [22,23,24]. Despite the wide tissue expression spectrum and the diversity of hormones it binds, the function of MC5R is not well addressed. So far, the known functions of MC5R are mainly involved in the regulations of exocrine gland function, energy, lipid metabolism and immune response [25,26,27]. A recent study shows that chicken MC5R has a higher affinity to ACTH/α-MSH, and is coupled to the cAMP/ protein kinase A (PKA) signaling pathway. As ACTH can promote gluconeogenesis and reduce triglyceride content in primary hepatocytes, it suggests that ACTH/MC5R may have an important role in regulating glucose and lipid metabolism in chicken liver [28].

Nonetheless, there are few studies on the role of MC5R in poultry nutrition and energy metabolism and the related mechanisms. The purpose of this study was to address the role and mechanisms of MC5R in the nutrition and energy metabolism of goose liver using the overfeeding model and the fasting/refeeding model, which may provide a theoretical reference for improving goose production performance by adjusting nutrition or energy levels.

## 2. Results

### 2.1. MC5R Expression in Different Tissues of Goose Embryos

**Figure 1:**
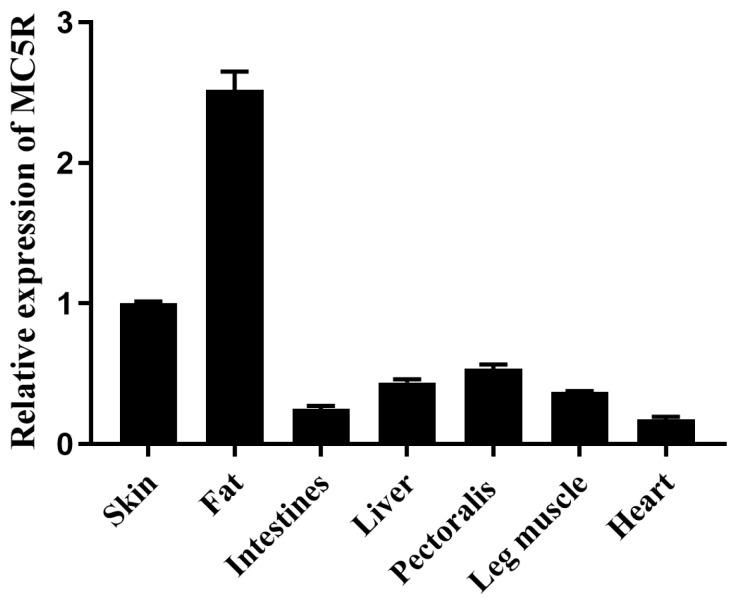
The mRNA expression level of *MC5R* gene in different embryonic tissues of Landes geese. Note: The mRNA expression level was determined by RT-qPCR and presented as fold change over skin tissue. The internal reference gene was glyceraldehyde-3-phosphate dehydrogenase (*GAPDH*). The embryos were from the fertilized eggs cultured for 23 days. n = 3.

The mRNA expression level of *MC5R* was determined in different tissues of Landes goose embryos (after 23 days of incubation) by quantitative real-time polymerase chain reaction (qPCR) analysis. The results showed that *MC5R* expression was the highest in fat tissue, followed by skin, liver and muscle tissues (pectoralis and leg muscles at a relatively higher level, while the intestine and heart had the lowest expression level) (Figure 1).

### 2.2. MC5R Expression of Goose Liver in the Overfeeding Model

**Figure 2:**
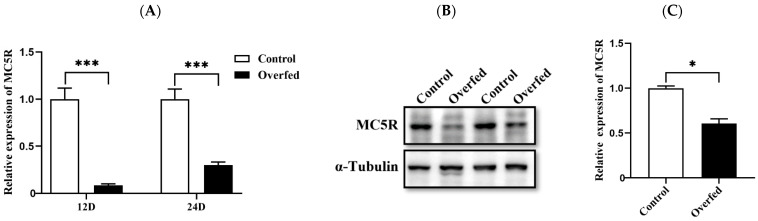
The mRNA and protein expression levels of *MC5R* in the livers of the overfed *versus* normally fed Landes geese on the 12th (12D) and 24th days (24D) of overfeeding. Note: (**A**) The mRNA expression level was determined by RT-qPCR and presented as fold change over the control group (the normally fed geese). The internal reference gene was *GAPDH*. n = 6. (**B**) The protein expression level was determined by immunoblot assay. The internal reference gene was α-tubulin. The protein samples were from the livers of geese on the 24th day of overfeeding. (**C**) Quantification of the immunoblots. *, *** indicate *p* < 0.05 and 0.001, respectively.

To investigate the role of MC5R in hepatic nutrition and energy metabolism, the expression of *MC5R* in the liver was determined in the Landes geese overfed for 12 and 24 days versus the normally fed geese (control). Quantitative PCR analysis indicated that the mRNA expression level of *MC5R* was significantly decreased in the overfed-induced fatty liver compared to normal liver (Figure 2A). Consistently, immunoblotting analysis showed that the protein expression level of MC5R was also significantly decreased in fatty liver vs. normal liver on the 24th day of overfeeding (Figure 2B).

### 2.3. MC5R Expression of Goose Liver in the Fasting/Refeeding Model

**Figure 3:**
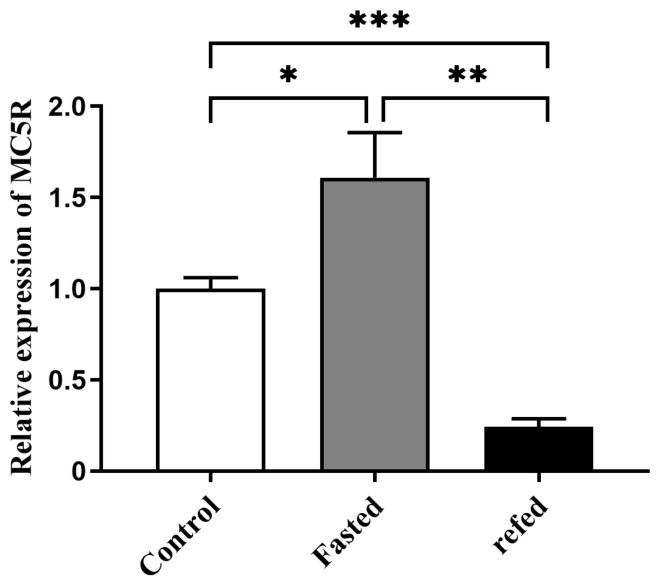
The mRNA expression level of *MC5R* gene in the livers of the fasted and refed *versus* control Landes geese. Note: The mRNA expression level was determined by RT-qPCR and presented as fold change over the control group. The internal reference gene was *GAPDH*. n = 8. *, **, *** indicate *p* < 0.05, 0.01 and 0.001, respectively.

Quantitative PCR analysis showed that the mRNA expression level of *MC5R* in goose liver after 24 h of fasting was significantly higher than that in goose liver of the control group (the normally fed geese), while this induction was significantly suppressed by 2 h of refeeding (Figure 3).

### 2.4. Effect of Nutrition or Energy Metabolism-Related Factors on MC5R Expression in Goose Hepatocytes

**Figure 4:**
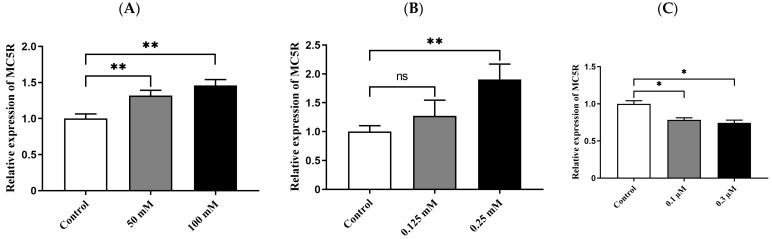
The mRNA expression level of *MC5R* in the treated versus control primary hepatocytes of goose. Note: The factors that are related to nutrition or energy metabolism include (**A**) glucose, (**B**) sodium oleate and (**C**) sodium thyroxine. The mRNA expression level was determined by RT-qPCR and presented as fold change over the control group. The internal reference gene was *GAPDH*. n = 6. *, ** indicate *p* < 0.05 and 0.01, respectively. ‘ns’ denotes *p* > 0.05.

After goose primary hepatocytes were treated with different concentrations of glucose, sodium oleate and sodium thyroxine for 14 h, the cells were harvested and used for determining the mRNA expression level of *MC5R* by qPCR analysis. The data showed that glucose at 50 mM and 100 mM and sodium oleate at 0.25 mM significantly induced *MC5R* expression compared with the control group (Figure 4A,B), while sodium thyroxine at 0.1 μM and 0.3 μM significantly inhibited *MC5R* expression (Figure 4C).

### 2.5. Effect of MC5R Overexpression on Downstream Gene Expression and Pathways in Goose Hepatocytes

**Figure 5:**
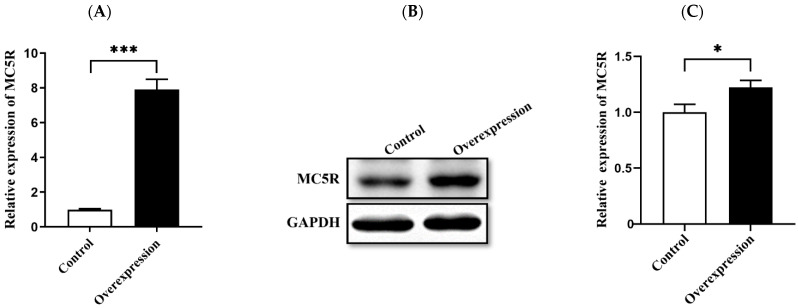
Overexpression of *MC5R* gene in goose primary hepatocytes. Note: The (**A**) mRNA and (**B**) protein expression levels of *MC5R* in goose primary hepatocytes treated with *MC5R* overexpression vector vs. empty vector (the control group). (**C**) Quantification of the immunoblots. The mRNA expression level was determined by RT-qPCR and presented as fold change over the control group. The internal reference gene was ubiquitin C (*UBC*). n = 6. The protein expression level was determined by immunoblot assay. The internal reference gene was *GAPDH*. *, *** indicate *p* < 0.05 and 0.001, respectively.

**Figure 6:**
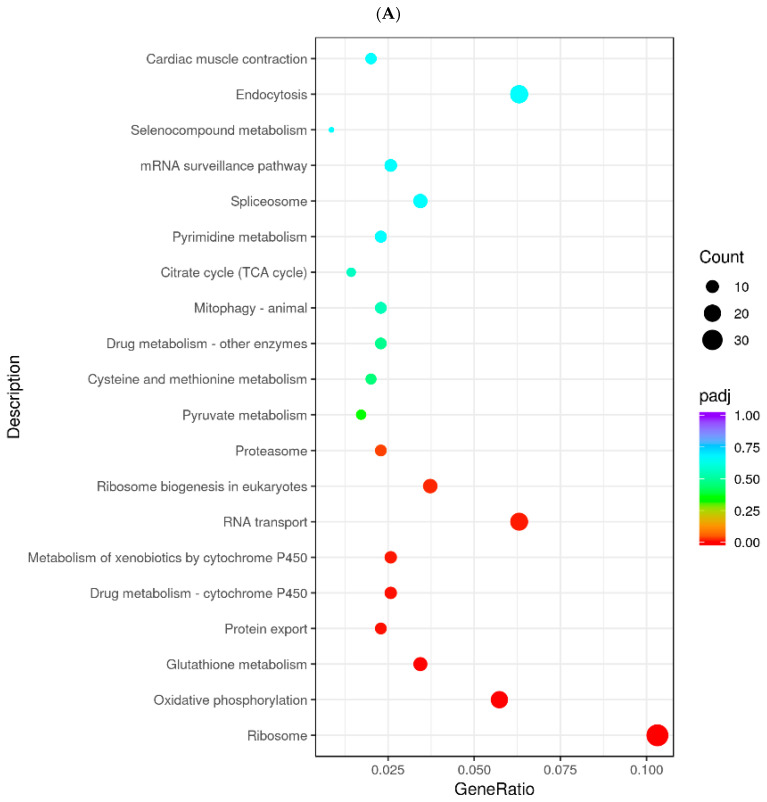

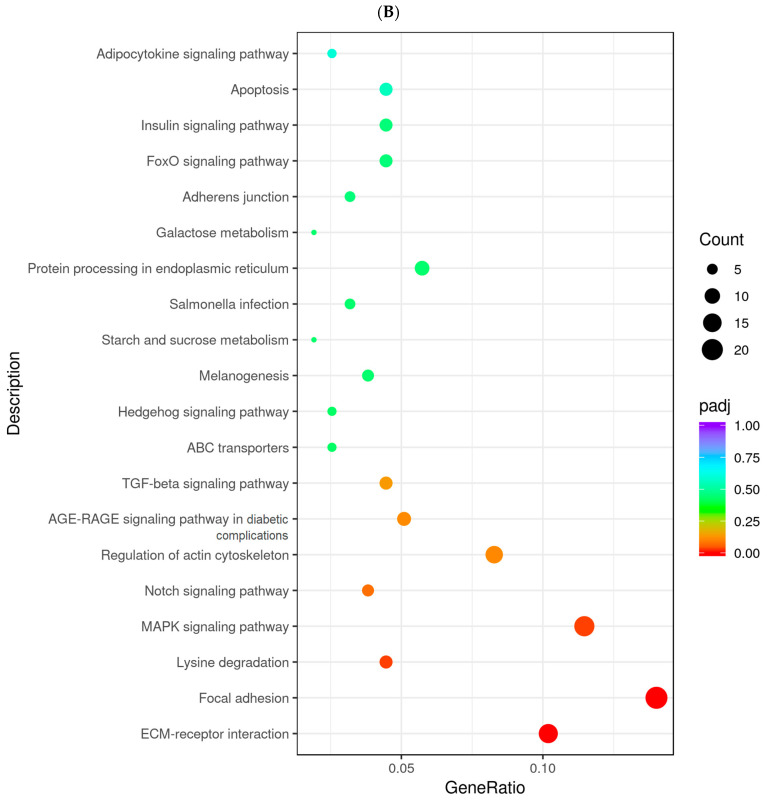
The dot chart shows the results from KEGG enrichment analysis. Note: (**A**) The results of KEGG enrichment analysis with the upregulated DEGs. (**B**) The results of KEGG enrichment analysis with the downregulated DEGs. The abscissa indicates the ratio of the number of the annotated DEGs in a specified KEGG pathway to the total number of annotated DEGs, and the ordinate indicates the KEGG pathways. The colors denote the adjusted *p*-value (*p*-adj), and the sizes of the dots denote the number of DEGs. The DEGs were identified in goose primary hepatocytes transfected with *MC5R* overexpression vectors vs. empty vectors (n = 4).

To identify the genes and pathways affected by *MC5R* in goose hepatocytes, *MC5R* was overexpressed in goose primary hepatocytes (Figure 5A,B), and transcriptome analysis was then performed. The data showed that *MC5R* overexpression significantly affected the expression of 1381 genes, of which the expression of 838 genes was induced by *MC5R* overexpression while that of 543 genes was suppressed (Supplementary Figure S1). According to the statistical significance (*p*-value), the top 10 up- and downregulated differentially expressed genes (DEGs) are listed in Table 1. Gene Ontology (GO) enrichment analysis showed that the upregulated DEGs were enriched in 604 GO terms, of which 322 terms belong to biological processes, 81 terms belong to cellular composition and 201 terms belong to molecular functions (Supplementary Figure S2A). In contrast, the downregulated DEGs were enriched in 381 GO terms, of which 150 terms belong to biological processes, 46 terms belong to cellular components and 185 terms belong to molecular functions (Supplementary Figure S2B). The Kyoto Encyclopedia of Genes and Genomes (KEGG) pathway analysis showed that the upregulated DEGs were mainly enriched in the ribosome, endocytosis, RNA transport, oxidative phosphorylation, ribosome biogenesis in eukaryotes, glutathione metabolism, spliceosome, metabolism of xenobiotics by cytochrome P450, cysteine and methionine metabolism, citrate cycle (TCA cycle) pathways, etc. (Figure 6A). In contrast, the downregulated DEGs were mainly enriched in the focal adhesion, MAPK signaling pathway, ECM–receptor interaction, regulation of actin cytoskeleton, protein processing in endoplasmic reticulum, AGE-RAGE signaling pathway in diabetic complications, FoxO signaling pathway, insulin signaling pathway, apoptosis, lysine degradation pathways, etc. (Figure 6B). To validate the results of the DEGs identified by transcriptome sequencing analysis, qPCR was used to determine the expression of 12 randomly selected DEGs. The results showed that among the 12 genes, only the lactate dehydrogenase B (*LDHB*) gene was not fully consistent with the RNA-seq data, which indicates the reliability of the RNA-seq analysis (Supplementary Figure S3).

### 2.6. Effect of MC5R Overexpression on the Expression of Some Specifically Selected Genes

**Figure 7:**
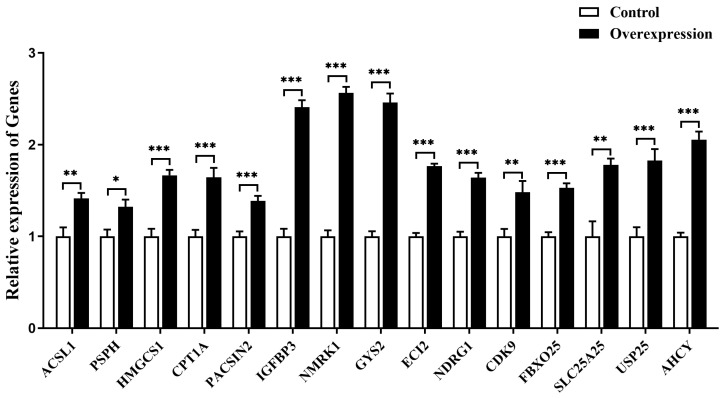
The mRNA expression level of some specifically selected DEGs in goose primary hepatocytes transfected with *MC5R* overexpression vector vs. empty vector. Note: The mRNA expression level was determined by RT-qPCR and presented as fold change over the control group. The internal reference gene was *UBC*. n = 6. *,**, *** indicate *p* < 0.05, 0.01 and 0.001, respectively.

Some downstream DEGs related to glycolipid metabolism (acyl-CoA synthetase long chain family member 1 (*ACSL1*), glycogen synthase 2 (*GYS2*), 3-hydroxy-3-methylglutaryl-CoA synthase 1 (*HMGCS1*), carnitine palmitoyltransferase 1A (*CPT1A*), enoyl-CoA delta isomerase 2 *(ECI2)*, nicotinamide riboside kinase 1 (*NMRK1*)), cell growth and death (insulin like growth factor binding protein 3 (*IGFBP3*), N-myc downstream regulated 1 (*NDRG1*), cyclin dependent kinase 9 (*CDK9*)) and other biological processes (phosphoserine phosphatase (*PSPH*), protein kinase C and casein kinase substrate in neurons 2 (*PACSIN2*), F-box protein 25 (*FBXO25*), solute carrier family 25 member 25 (*SLC25A25*), ubiquitin specific peptidase 25 (*USP25*) and adenosylhomocysteinase (*AHCY*)) are specifically selected. The expression of these genes in goose primary hepatocytes transfected with *MC5R* overexpression vectors vs. empty vectors was determined by qPCR. The data showed that *MC5R* overexpression significantly induced the expression of these genes in goose hepatocytes (Figure 7).

### 2.7. Effects of MC5R Knockdown on the Expression of Some Specifically Selected Genes

**Figure 8:**
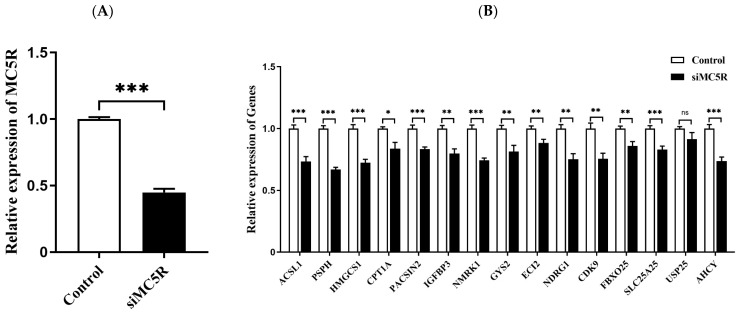
The mRNA expression level of *MC5R* (**A**) and some specifically selected DEGs (**B**) in goose primary hepatocytes transfected with siRNA targeting MC5R (siMC5R) versus the control negative siRNA (control). Note: The mRNA expression level was determined by RT-qPCR and presented as fold change over the control group. The internal reference gene was *UBC*. n = 6. *,**, *** indicate *p* < 0.05, 0.01 and 0.001, respectively. ‘ns’ denotes *p* > 0.05.

To validate these downstream genes of *MC5R*, goose primary hepatocytes were transfected with siRNAs targeting *MC5R* (si*MC5R*) or negative control siRNAs. The expression of the downstream genes in these hepatocytes was then determined by qPCR analysis. The data showed that the expression of *MC5R* was successfully knocked down by *MC5R* siRNAs (Figure 8A), and the mRNA expression of the downstream genes except for the *USP25* gene was significantly suppressed by *MC5R* knockdown (Figure 8B).

### 2.8. Effect of Glucose on the Expression of Some Specifically Selected Genes in Goose Hepatocytes

**Figure 9:**
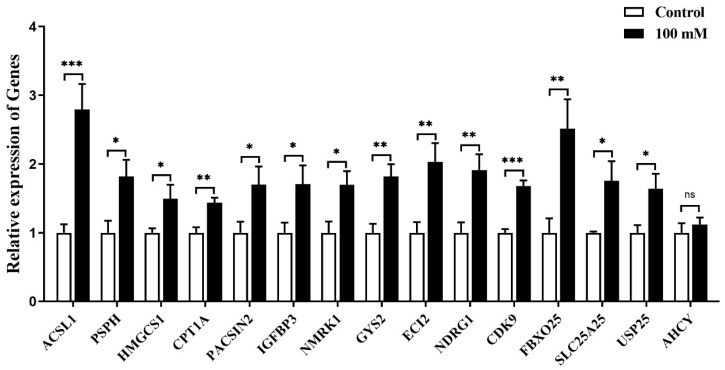
The mRNA expression level of some specifically selected DEGs in goose primary hepatocytes treated with 100 mM/L glucose versus the control group (0 mM/L glucose). Note: The mRNA expression level was determined by RT-qPCR and presented as fold change over the control group. The internal reference gene was *GAPDH*. n = 6. *,**, *** indicate *p* < 0.05, 0.01 and 0.001, respectively. ‘ns’ denotes *p* > 0.05.

As glucose at the concentration of 100 mmol/L could induce the expression of *MC5R* in goose primary hepatocytes, the expression of the specifically selected genes was determined in the glucose-treated cells versus the untreated control cells by qPCR analysis. The results showed that the mRNA expression of *ACSL1*, *PSPH*, *HMGCS1*, *CPT1A*, *PACSIN2*, *IGFBP3*, *NMRK1*, *GYS2*, *ECI2*, *NDRG1*, *CDK9*, *FBXO25*, *SLC25A25* and *USP25* genes was significantly induced by glucose treatment, except for *AHCY* gene (Figure 9).

### 2.9. Effect of Overfeeding on the Expression of Some Specifically Selected Genes in Goose Liver

**Figure 10:**
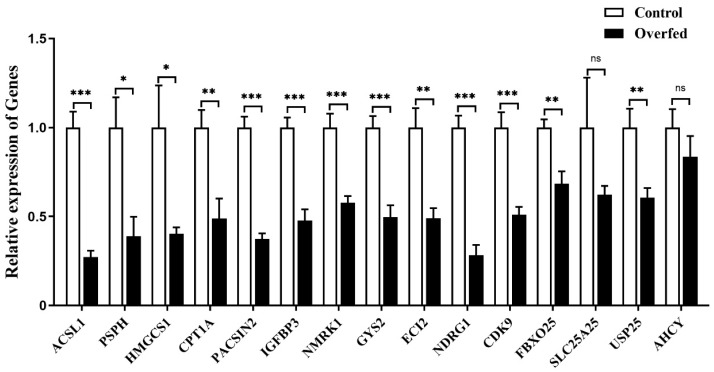
The mRNA expression level of some specifically selected DEGs in the livers of the overfed versus normally fed geese on the 24th day of overfeeding. Note: The mRNA expression level was determined by RT-qPCR and presented as fold change over the control group (the normally fed geese). The internal reference gene was *GAPDH*. n = 6. *,**, *** indicate *p* < 0.05, 0.01 and 0.001, respectively. ‘ns’ denotes *p* > 0.05.

Quantitative PCR analysis showed that the expression of the downstream genes was significantly suppressed in goose liver by overfeeding, except for *SLC25A25* and *AHCY* genes (Figure 10).

### 2.10. Effects of Refeeding on the Expression of Some Specifically Selected Genes in Goose Liver

**Figure 11:**
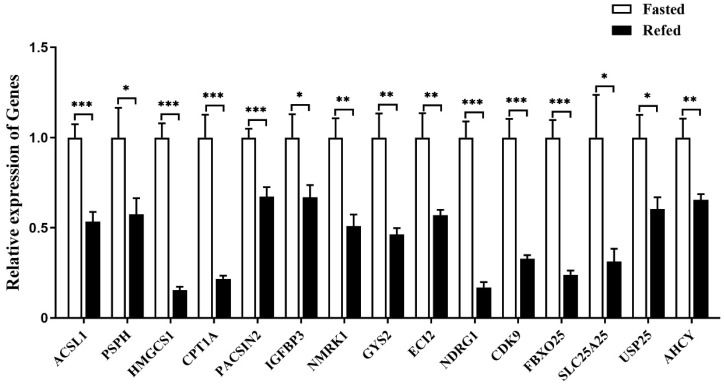
The mRNA expression level of some specifically selected DEGs in the livers of the refed versus fasted geese. Note: The mRNA expression level was determined by RT-qPCR and presented as fold change over the fasted group. The internal reference gene was *GAPDH*. n = 8. *,**, *** indicate *p* < 0.05, 0.01 and 0.001, respectively.

Quantitative PCR analysis showed that the expression of all the selected downstream genes in goose liver was significantly suppressed by refeeding compared with the fasting group (Figure 11).

### 2.11. PPI Analysis of Selected Downstream Genes

**Figure 12:**
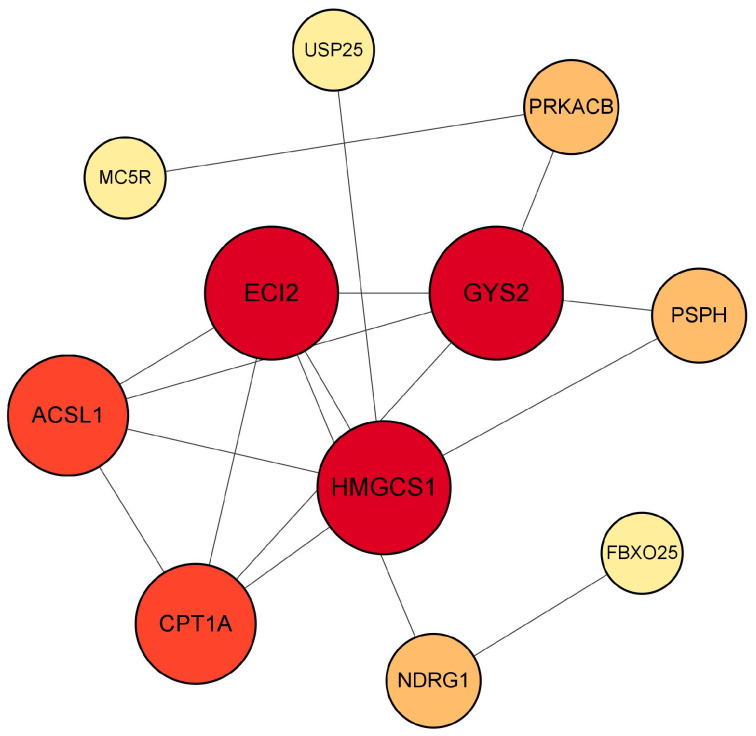
The chart shows the protein–protein interaction network regulated by *MC5R* based on PPI analysis. Note: Colors ranging from yellow to red indicate an increase in degree of connectivity, and a larger degree indicates a greater connectivity to other genes.

As it is known that MC5R can exert a variety of biological functions through the PKA signaling pathway, *PKA*, *MC5R* and its downstream genes, including *ACSL1*, *PSPH*, *HMGCS1*, *CPT1A*, *PACSIN2*, *IGFBP3*, *NMRK1*, *GYS2*, *ECI2*, *NDRG1*, *CDK9*, *FBXO25*, *SLC25A25* and *USP25*, were input into the STRING database for protein–protein interaction network prediction. The result showed that *PKA*, *GYS2*, *ECI2*, *PSPH*, *CPT1A*, *ACSL1*, *HMGCS1*, *USP25* and *NDRG1* participated in the network regulated by MC5R (Figure 12). It is worth mentioning that *GYS2* had the highest degree of connectivity to other genes, suggesting that this gene was the key gene mediating the role of MC5R in the regulation of energy metabolism of goose liver.

## 3. Discussion

The level of nutrition or energy metabolism has a great impact on the growth, health and production performance of animals, and the liver plays an important role in nutrition or energy metabolism. In this study, the data from the in vivo experiments showed that increasing the level of nutrition or energy (refeeding or overfeeding) could inhibit the expression of *MC5R* in goose liver and vice versa. Refeeding or overfeeding usually elevates the levels of glucose, insulin, thyroxine and other molecules in the blood, which may inhibit the expression of *MC5R* and its downstream genes involved in fatty acid oxidation and lipolysis, thus promoting fat deposition in the liver. On the other hand, fasting usually lowers the levels of glucose, insulin and thyroxine in the blood and elevates the levels of glucagon and other molecules in the blood, which may induce the expression of *MC5R* and its downstream genes, thus promoting gluconeogenesis and fat mobilization in the liver. In a word, changes in nutrition and energy levels can alter the expression levels of *MC5R* and its downstream genes, through which the energy fuel in animals switches from fat to sugar during feed intake and from sugar to fat during fasting.

Different from the in vivo experiments, the expression of *MC5R* in goose primary hepatocytes was induced by increased nutrition or energy levels, such as the elevated concentrations of glucose or oleic acid. This inconsistency indicates that in addition to the direct effect on the expression of *MC5R* in goose liver, the level of nutrition or energy in vivo may also indirectly affect the expression of *MC5R* by other factors. For example, short-term fasting can significantly reduce the level of thyroid hormone in chicken blood [29,30]. In this study, thyroxine treatment inhibited the expression of *MC5R* in goose primary hepatocytes, suggesting that changes in the nutrition and energy levels may indirectly regulate the expression of *MC5R* in goose liver by influencing the level of thyroid hormone in the blood. In addition, there are a lot of interactions in vivo among different types of cells and multiple systems, which is much more complicated than the in vitro situation of the cultured cells. This may provide another explanation for the inconsistency mentioned above.

There are a number of previous studies that report the close relationship of MC5R with the nutrition or energy metabolism of animals. Firstly, in mammals, MC5R is not only expressed in the skin, adrenal gland, testis and heart, but also highly expressed in the tissues closely related to nutrition and energy metabolism, i.e., fat, liver, brain and muscle [31,32]. Similar to mammals, MC5R is expressed in diverse tissues of chickens, such as the liver, adrenal gland, ovary and adipose tissue [33]. In this study, *MC5R* was expressed in skin, fat, small intestine, liver, pectoralis, leg muscle and heart tissue of 23-day-old Landes goose embryos, and its expression was relatively higher in skin, fat, liver and skeletal muscle. Secondly, the expression of *MC5R* is significantly correlated with body mass index, subcutaneous fat, adiposity and resting metabolic rate [23]. In addition, *MC5R* knockdown leads to decreased fat amount in the sebum of mice [34]. Therefore, it is believed that MC5R is involved in nutrition and energy metabolism, especially lipid metabolism. Considering the results from transcriptome analysis on goose liver in the fasting and refeeding experiment, that is, fasting mainly affected lipid metabolism in goose liver while refeeding affected both lipid metabolism and glucose and amino acid metabolism [11], we speculate that MC5R may play a role in regulating lipid metabolism in the process of nutrition and energy metabolism.

Moreover, activated MC5R can stimulate lipid mobilization in adipocytes and glucose uptake in skeletal muscle [35]. It has been shown that the binding of α-MSH to MC5R activates the PKA and ERK1/2 (MAPK) signaling pathways, and the activation of MC5R in skeletal muscle regulates fatty acids oxidation in muscle cells through PKA and MAPK signaling pathways [36]. It has also been shown that in HEK293 cells, MC5R activates the ERK1/2 signaling pathway via phosphatidylinositol 3-kinase (PI3K), and this activation is not dependent on adenylate cyclase, PKA, protein kinase C (PKC) and akt kinase (Akt) / protein kinase B (PKB) pathways [37]. Consistently, transcriptome analysis of goose primary hepatocytes overexpressing *MC5R* in this study showed that some of the DEGs were enriched in the signaling pathways related to glucolipid metabolism, including oxidative phosphorylation, citrate cycle, MAPK signaling pathway, adipocytokine signaling pathway, insulin signaling pathway, pyruvate metabolism, FoxO signaling pathway, starch and sucrose metabolism, arachidonic acid metabolism and galactose metabolism. Additionally, in this study, some downstream genes involved in the regulation of goose liver energy metabolism were identified using fasting/refeeding model, overfeeding model and glucose-treated goose hepatocytes, and using these genes, PPI analysis suggested that *MC5R* could regulate the protein–protein interaction network consisting of the downstream genes, including *GYS2*, *ECI2*, *PSPH*, *CPT1A*, *ACSL1*, *HMGCS1*, *USP25* and *NDRG1*, through *PKA*, and *GYS2* might be a key gene mediating the role of MC5R in the regulation of energy metabolism of goose liver. The genes associated with glycolipid metabolism, including *ACSL1*, *CPT1A*, *GYS2*, *HMGCS1* and *ECI2,* were affected by changes in nutrition or energy level in the fasting/refeeding model, the overfeeding model and glucose-treated goose hepatocytes. Interestingly, the correlation between the expressions of these genes and *MC5R* is in line with the notion that the expression of these genes is regulated by MC5R. This provides more solid evidence supporting the conclusion that MC5R mediates the biological effects of nutrition and energy metabolism mainly by influencing glycolipid metabolism.

It is noteworthy that the expression of *ACSL1*, *CPT1A*, *GYS2*, *HMGCS1* and *ECI2* genes in goose fatty liver was inhibited by overfeeding, and this inhibition by high levels of nutrition and energy has also been reported in previous studies. For example, in an animal model of high-fat-induced obesity, the expression of *ACSL1* and *HMGCS1* was inhibited in both hamster and zebrafish livers, and steatosis also occurred in the livers of these animals [38,39]. It has also been reported that inhibition of *ECI2* expression results in decreased glucose utilization and increased fat deposition [40].

In addition to being engaged in the regulation of glycolipid metabolism, the transcriptome analysis in this study also revealed that MC5R was involved in amino acid metabolism (cysteine and methionine metabolism, lysine degradation), ECM–receptor interaction, the AGE-RAGE signaling pathway, the notch signaling pathway and the glutathione metabolism pathway. The expression level of some DEGs involved in these pathways, such as *PSPH*, *PACSIN2*, *IGFBP3*, *NMRK1*, *NDRG1*, *CDK9*, *FBXO25*, *SLC25A25*, *USP25* and *AHCY* genes, is correlated with the expression level of *MC5R* in the fasting/refeeding model, the overfeeding model and the glucose-treated goose hepatocytes. This to a large extent agrees with the speculation that these genes are regulated by *MC5R*. Among these genes, it is known that *IGFBP3*, *NDRG1* and *CDK9* genes can regulate cytokine expression, cell growth and apoptosis [41,42,43].

In summary, the expression of *MC5R* in goose hepatocytes is influenced by changes in the level of nutrition or energy metabolism, and glucose, oleic acid and thyroxine, may be involved in the regulation of *MC5R* expression in goose hepatocytes. MC5R can mediate the biological effects caused by changes in the level of nutrition or energy metabolism in goose hepatocytes through multiple pathways, especially glycolipid-metabolism-related pathways.

## 4. Materials and Methods

### 4.1. Animal Welfare Statement

The animal protocols in this study were approved by the Institutional Animal Care and Use Committee (IACUC) of Yangzhou University, and all animal experiments were performed according to the approved protocols so that animal pain and suffering were minimized as much as possible. The certificate number assigned by IACUC is SYXK(Su)2016-0020.

### 4.2. Experimental Animals and Sample Collection

To determine the expression profile of *MC5R* in different goose tissues, 10 fertilized goose eggs were incubated at 37.8 °C. After 23 days of incubation, the embryos were sacrificed, and the tissues, including pectoralis, leg muscles, heart, liver, small intestine, fat and skin tissues, were collected and stored at −70 °C.

For the animal fasting and refeeding experiment, 24 10-day-old Landes goslings with similar body weight were randomly divided into three groups (n = 8), including the control group, the fasting group and the refeeding group. The geese in the control group had free access to feed and water, those in the fasting group were fasted for 24 h with free access to water, and those in the refeeding group were fasted for 24 h with free access to water, followed by refeeding for 2 h with free access to feed and water. After that, the goslings were sacrificed, and the liver was collected and stored at −70 °C.

For the animal overfeeding experiment, 24 65-day-old male Landes geese with similar body weight raised in Licheng Livestock & Poultry Co., Ltd., Huai’an, China were randomly assigned into two groups (n = 12), i.e., the control group and the overfeeding group. The geese in the control group were fed ad libitum, and those in the overfeeding group were artificially overfed for 24 days. The overfeeding protocol has been described previously [44]. The two groups were fed the same diet. On the 12th and 24th days of overfeeding, 6 geese from each group were sacrificed, and the liver tissues were collected and stored at −70 °C.

### 4.3. Isolation, Culture and Treatment of Goose Primary Hepatocytes

After the fertilized goose eggs were incubated for 23 days, the embryos were sacrificed for liver tissue collection. According to the protocol described previously [45], primary hepatocytes were prepared and plated at a density of 5 × 10^6^ cells per well. The cells were incubated overnight in a complete culture medium containing high-glucose DMEM culture medium (Gibco, New York, NY, USA), 10% fetal bovine serum (Gibco, New York, NY, USA), 1% penicillin–streptomycin (Solarbio, Beijing, China) and 0.1% EGF (Solarbio, Beijing, China). For glucose treatment, the cells in complete culture medium were treated with 0 mmol/L (the control group), 50 mmol/L and 100 mmol/L glucose, respectively. For oleate treatment, the cells in complete culture medium were treated with 0 mmol/L (the control group), 0.125 mmol/L and 0.25 mmol/L sodium oleate, respectively. For thyroxine treatment, the cells in complete culture medium were treated with 0 µmol/L (the control group), 0.1 µmol/L and 0.3 µmol/L sodium thyroxine, respectively. After 14 h of treatment, the cells were collected for later analysis.

### 4.4. MC5R Overexpression and Knockdown in Goose Primary Hepatocytes

For *MC5R* overexpression, pcDNA3.1 was used as the overexpression vector. For *MC5R* knockdown, and siRNA (si*MC5R*) targeting *MC5R* (sense: 5′-GGCUCCAUUCUUUCCUCCAUTT-3′, antisense: 5′-AUGGAGGAAGAAUGGAGCCTT-3′) and the negative control (sense: 5′-UUCUCCGAACGUGUCACGUTT-3′, antisense: 5′-ACGUGACACGUUCGGAGAATT-3′) were purchased from Suzhou Jima Biotechnology Co. (Suzhou, China). For transcription, *MC5R* overexpression vector, empty vector (pcDNA3.1), si*MC5R*, and negative siRNA were separately transfected into goose primary hepatocytes using jetPRIME^®^ transfection reagent (Polyplus, Illkirch, France) according to the manufacturer’s instructions. The cells were harvested after 36 h of transfection.

### 4.5. Quantitative PCR Analysis

Total RNA was extracted from tissue samples or cells using RNA-easy Isolation Reagent (Vazyme, Nanjing, China) according to the manufacturer’s instructions. The complementary DNA (cDNA) was synthesized using a HiScript III RT SuperMix for qPCR (+gDNA wiper) Reverse Transcription Kit (Vazyme, Nanjing, China). Quantitative PCR was performed using the AceQ qPCR SYBR Green Master Mix kit (Vazyme, Nanjing, China) according to the procedures previously described [46]. Glyceraldehyde-3-phosphate dehydrogenase (*GAPDH*) and ubiquitin C (*UBC*) were used as the internal reference genes. The relative expression of the gene of interest was calculated using the 2^−ΔΔCt^ method [47]. The primers used in qPCR analysis were designed with Primer 5.0 software using the reference sequence of each deposited in the National Center of Biotechnology Information (NCBI) database (Table S1) and synthesized by Azenta Life Science and Technology Co. (Nanjing, China).

### 4.6. Immunoblot Analysis

The collected tissue and cell samples were lysed using RIPA lysis solution (Solarbio, Beijing, China), and protein concentration of each sample was determined using a BCA Protein Assay Kit (Beyotime, Shanghai, China). The samples were then mixed with 5× SDS–PAGE loading buffer (NCM Biotech, Suzhou, China) and heated at 100 °C for 5 min. After sample preparation, 20 μg protein sample per well was loaded and separated on a 12% BeyoGel SDS–PAGE Precast Gel (Beyotime, Shanghai, China) by electrophoresis, which was followed by transferring to 0.45 µm PVDF membrane (Millipore, Burlington, MA, USA). After blocking with a commercial Fast Blocking Solution (NCM Biotech, Suzhou, China) for 20 min, the membrane was incubated with the primary antibody (1:1000) overnight at 4 °C, followed by incubating the membrane with the secondary antibody (polyclonal sheep anti-mouse or sheep anti-rabbit 1:10,000) for 1 h at room temperature. The immunoblots were visualized using a Tanon development instrument (Tanon, Shanghai, China). The primary antibodies include MC5R antibody (Cat No.: MAB8205, R&D Systems, Minneapolis, MN, USA), α-Tubulin (Cat No.: 2144; Cell Signaling Technology, Danvers, CO, USA) and GAPDH (Cat No.: bsm-33033M; Bioss, Beijing, China).

### 4.7. Transcriptome Sequencing Analysis

Total RNA was extracted from cell samples using TRIzol reagent according to the manufacturer’s instructions, then mRNA was purified from total RNA using poly-T oligo-attached magnetic beads. The purified mRNA was fragmented and used for cDNA synthesis. The quality and quantity of the synthesized cDNA were determined, followed by construction of the cDNA library. The cDNA library was sequenced on the Illumina platform using the PE150 sequencing strategy. Junctions and low-quality reads were removed from the raw data. Q20 and Q30 were calculated and applied to the clean data for checking the sequencing error rate. The clean reads were de novo assembled using HISAT2 (v2.0.5) software, and the expression level of genes was calculated using the fragments per kilobase per million fragments (FPKM) method. Differential expression analysis between groups was performed by the DESeq2 method, and then adjusted *p*-value (*p*-adj) was calculated using Benjamini and Hochberg methods. The criteria for differentially expressed genes (DEGs) is *p*-adj < 0.05. At last, the DEGs were functionally annotated, followed by Gene Ontology (GO) enrichment analysis and Kyoto Encyclopedia of Genes and Genomes (KEGG) pathway enrichment analysis using the ClusterProfiler R package. The high-throughput transcriptome sequencing analysis and data analysis were performed by Beijing Novogene Technology Co., Ltd. (Beijing, China).

### 4.8. PPI Analysis

The protein–protein interaction network regulated by *MC5R* was constructed by inputting a list of known (*PKA*) and selected downstream genes of *MC5R* into a STRING database (https://cn.string-db.org/ (accessed on 23 March 2023)).

### 4.9. Statistical Analysis

Data are expressed as the mean ± standard error. The statistical significance of the difference between the two groups was determined using the *t* test (two-tailed), and that of the difference among the groups more than two was determined using one-way ANOVA. Statistical significance was established a priori as *p* < 0.05.

## Figures and Tables

**Table 1:** Top 10 up- and downregulated differentially expressed genes with the lowest *p* values.

Gene	Log_2_(Fold Change)	*p*-Value	Function Annotation
Upregulated			
*TM4SF4*	0.347	2.70 × 10^−16^	Involved in the regulation of cell proliferation, growth, development, activation and motility
*HPGD*	0.372	2.43 × 10^−9^	Responsible for metabolism of prostaglandins, oxidating hydroxylated polyunsaturated fatty acids, yielding keto metabolites
*EMC2*	0.335	4.21 × 10^−9^	Involved in the cotranslational insertion of multi-pass membrane proteins, regulating the insertion of various proteins in membranes
*ANXA8L1*	0.338	4.69 × 10^−9^	Inhibiting the thromboplastin-specific complex as an anticoagulant indirectly, involved in prostaglandin synthesis and regulation
*SERPINB5*	0.322	5.39 × 10^−9^	Involved in negative regulation of endopeptidase activity, extracellular matrix organization, prostate gland morphogenesis, regulation of epithelial cell proliferation
*EIF2S1*	0.335	5.71 × 10^−9^	Regulating protein synthesis initiation
*LOC106048698*	0.230	6.07 × 10^−9^	Unknown
*TMEM247*	0.406	1.33 × 10^−8^	Active in endoplasmic reticulum, regulating skin pigmentation
*TCP1*	0.286	2.33 × 10^−8^	Assisting proteins folding, regulating telomere maintenance
*NSUN2*	0.300	3.08 × 10^−8^	Involved in epidermal stem cell differentiation, testis differentiation and maternal to zygotic transition
Downregulated			
*A2M*	−0.588	1.49 × 10^−12^	Inhibiting a broad spectrum of proteases and inflammatory cytokines
*LOC106041921*	−0.406	8.21 × 10^−10^	Unknown
*COL1A2*	−0.505	2.89 × 10^−9^	Involved in collagen chain trimerization, identical protein binding and protein–macromolecule adaptor activity
*ADAMTS9*	−0.475	3.65 × 10^−9^	Involved in cleavage of proteoglycans, control of organ shape, inhibition of angiogenesis
*LOC106048083*	−0.948	3.81 × 10^−9^	Unknown
*COL6A3*	−0.695	7.01 × 10^−9^	Involved in collagen chain trimerization, serine-type endopeptidase inhibitor activity
*A2ML1*	−0.586	1.70 × 10^−8^	Inhibiting several proteases, forming covalent interactions with proteases, involved in serine-type endopeptidase inhibitor activity and peptidase inhibitor activity
*CYGB*	−0.333	3.37 × 10^−8^	Involved in oxygen transport, protection from oxidative stress, metabolism of nitric oxide, iron ion binding and oxygen binding
*IGSF3*	−0.504	6.08 × 10^−8^	Regulating cell cycle and promoting tumor development
*FBN1*	−0.489	6.22 × 10^−8^	Regulating glucose homeostasis

Note: The differentially expressed genes were identified in goose primary hepatocytes transfected with *MC5R* overexpression vectors versus empty vectors.

## Data Availability

The raw sequence data reported in this paper have been deposited in the Genome Sequence Archive (Genomics, Proteomics & Bioinformatics 2021) in National Genomics Data Center (Nucleic Acids Res 2022), China National Center for Bioinformation/Beijing Institute of Genomics, Chinese Academy of Sciences (GSA: CRA009202) that are publicly accessible at https://ngdc.cncb.ac.cn/gsa (accessed on 21 December 2022).

## Supplementary Materials

The following supporting information can be downloaded at: https://www.mdpi.com/article/10.3390/ijms24108648/s1.
