# Supplementary material for: Study on the Mechanism of MC5R Participating in Energy Metabolism of Goose Liver
Source: Int J Mol Sci. 2023 May 12;24(10):8648. doi: 10.3390/ijms24108648 (PMC10218275; doi:10.3390/ijms24108648)
Supplement: Supplementary file 1 [file ijms-24-08648-s001.zip › ijms-2340763-supplementary.pdf]

Supplementary Material

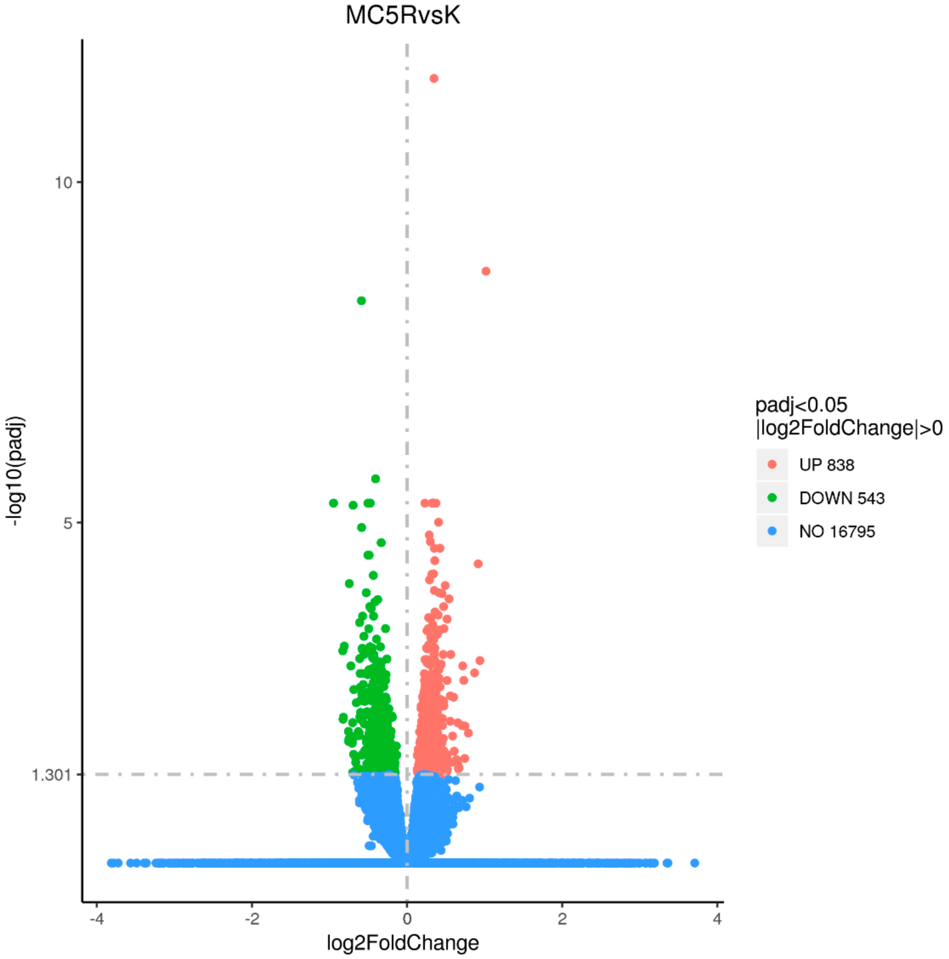

Supplementary Figure S1. Volcano plot showing the distribution of the differentially expressed genes (DEGs) identifed by transcriptome analysis. Note: The red dots are the upregulated DEGs identified in goose primary hepatocytes treated with *MC5R* overexpression vector vs. empty vector. The green dots are the downregulated DEGs. The blue dots are the genes whose mRNA expression level is not significantly different between the hepatocytes treated with *MC5R* overexpression vector and empty vector.

A

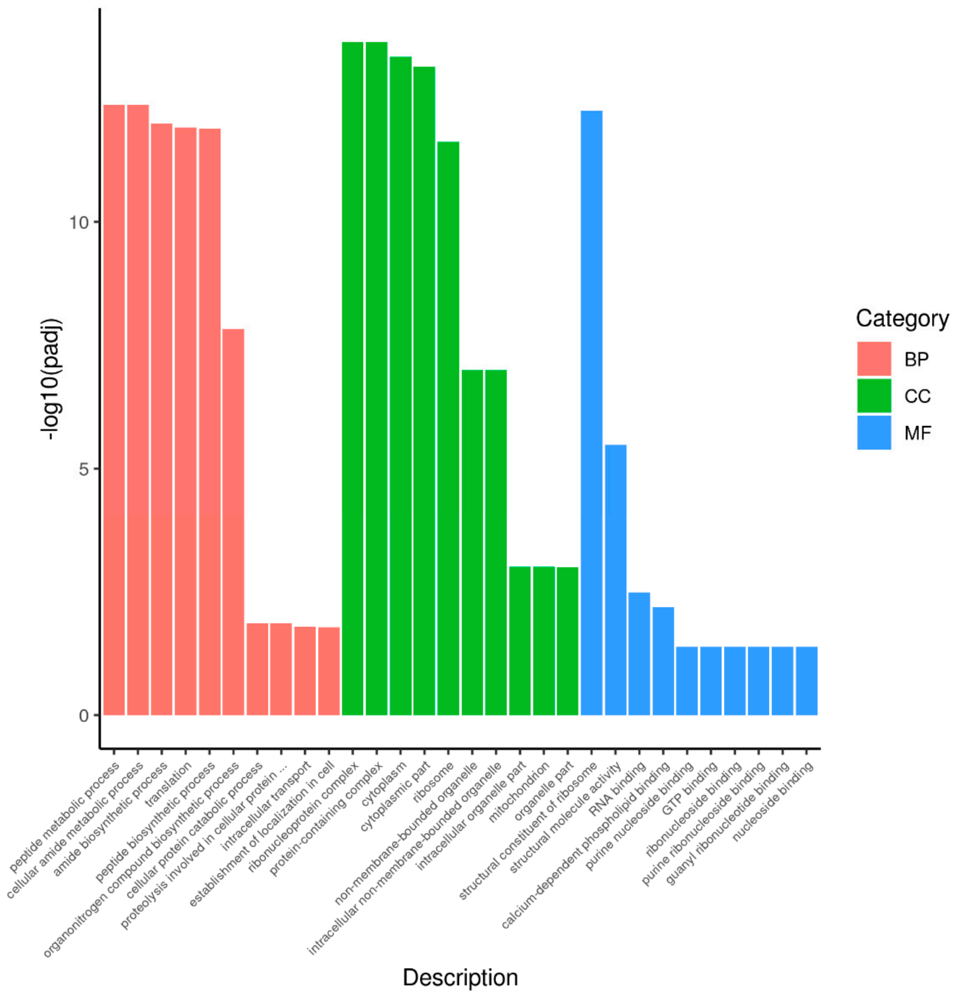
-log10(padj)
0
5
10
Description
peptide metabolic process
cellular amide metabolic process
amide biosynthetic process
translation
peptide biosynthetic process
organonitrogen compound biosynthetic process
cellular protein catabolic process
proteolysis involved in cellular protein ...
intracellular transport
establishment of localization in cell
ribonucleoprotein complex
protein-containing complex
cytoplasm
cytoplasmic part
ribosome
non-membrane-bounded organelle
intracellular non-membrane-bounded organelle
intracellular organelle part
mitochondrion
organelle part
structural constituent of ribosome
structural molecule activity
RNA binding
calcium-dependent phospholipid binding
purine nucleoside binding
GTP binding
ribonucleoside binding
purine ribonucleoside binding
guanyl ribonucleotide binding
nucleoside binding
Category
BP
CC
MF

B

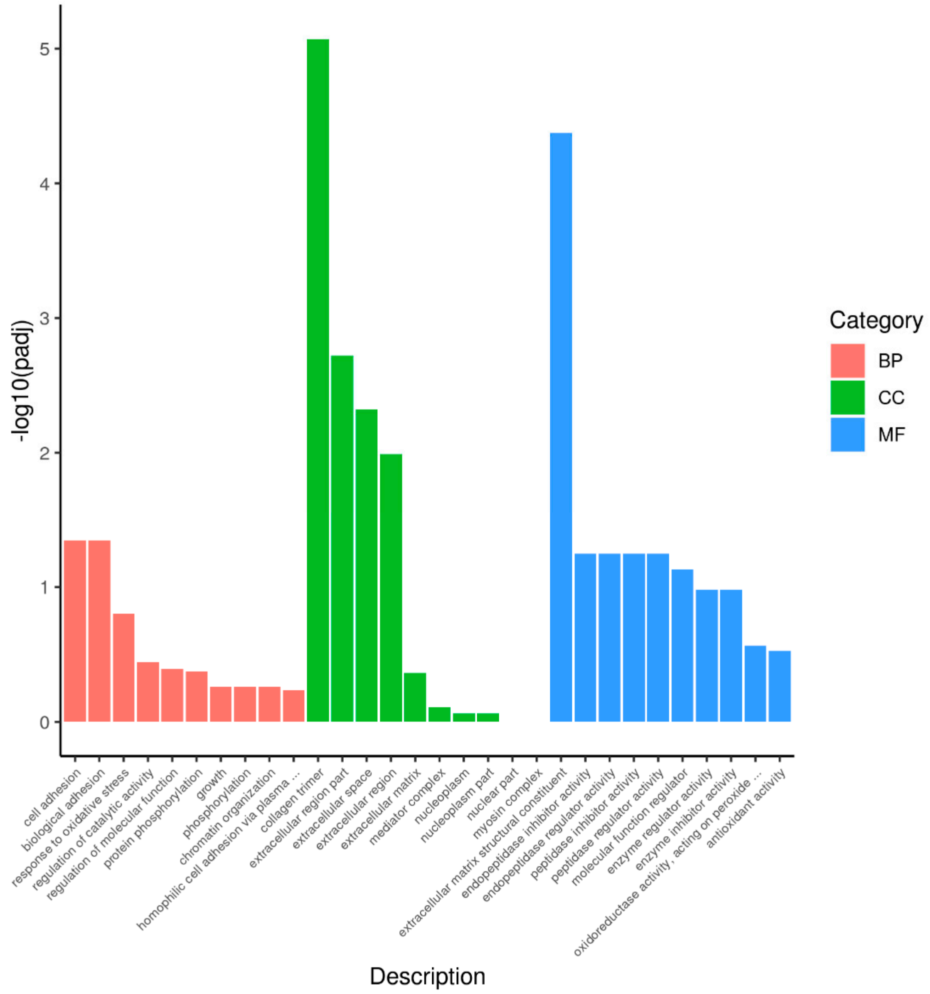

Supplementary Figure S2. The top 20 Gene Ontology (GO) terms that are enriched with the upregulated (A) or downregulated (B) differentially expressed genes (DEGs). Note: The DEGs are identified in goose primary hepatocytes treated with *MC5R* overexpression vector versus empty vector.

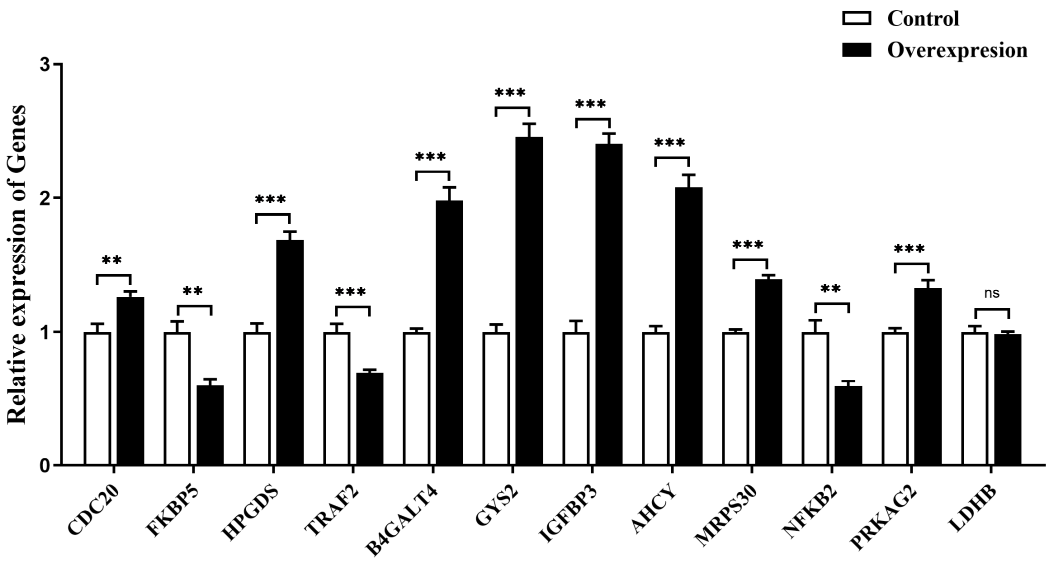

Supplementary Figure S3. Validation of some randomly selected differentially expressed genes (DEGs) identified from RNA-seq analysis. Note: The DEGs are identified in goose primary hepatocytes treated with *MC5R* overexpression vector versus empty vector. The mRNA expression level was determined by RT-qPCR and presented as fold change over the control group. The internal reference gene was *UBC*. n = 6. **, *** indicate $p < 0.01$ and 0.001, respectively. ‘ns’ denotes $p > 0.05$.

Supplementary Table

Table S1. The sequences of primers used for qPCR analysis

Gene	forward (5’-3’)	reverse (5’-3’)
MC5R	F: GCCTGGGTACAACTCTGTCC	R: AGCCCAGCAAACGATGAAGA
GAPDH	R: GCCATCAATGATCCCTTCAT	R: CTGGGGTCACGCTCCTG
UBC	F: AGGGTGGATTCTTTCTGG	R: ACTGAGTTTGGAGGGAGC
AHCY	F: TTCAGGGCAGACACTTCGAG	R: TGTGACGGCCATTCCTCAAT
CDC20	F: GCGCTGTTGATGCTCATTCC	R: CCGATGCCACTGTTGAGCTA
TRAF2	F: GGATGTGCTGAGTTGGTGGA	R: GGAAAGGGCAGGAAGATGCT
HPGDS	F: TAACAAATGGCTGGTGGGGA	R: GCCAAAAGCCTGGGGTACTT
IGFBP3	F: TCATCAGGAAAGAGCAAGCCA	R: CCTTTGGATGGACGACATTGC
FKBP5	F: TCGGGGACAAGGTTTACGTG	R: CACACCTCGCCCTTCTTCAT
B4GALT4	F: GGGTCTGATGGTGCTATGGG	R: CCTCGCAGGTATGGAGACAC
GYS2	F: ACCCATGCCACTCTTCTTGG	R: AGCCATTTGGGGTGACAACA
MRPS30	F: ATGATAAGCCGCACCTCCAG	R: GGCAGTTTGGACGGTTTGTG
NFkB2	F: GATCGACGTGGTCGTTAGCA	R: TCACCATCTCTTCATCGCCC
LDHB	F: TTGTGCCGGGTTCATTCAGT	R: TTAGGACACAAGGCAGGCTC
PRKAG2	F: CGGACACAGGGCAGAGATTT	R: AGGTAGCACAGGGAGATGGT
ACSL1	F: ACACACCTGACAAGGCCAAA	R: GGGTTTCCTGTCGTTCCACT
PSPH	F: CCTCAGCTAACGCCAGGAAT	R: ACCCCCAGAGACCAAGAAGA
HMGCS1	F: GACCAGAACCCAGAGATGGC	R: GGCTAGAAGAGAGGCAAGGC
CPT1A	F: ATGACTGGTGCTGGCATTGA	R: GCCAGCATCTCAGGGTTCTT
PACSIN2	F: CTACCCCACAGATTGGTCCG	R: ACTCTCACTTCCATTGCGGG
NMRK1	F: ACACCGGGGTACTTTGATGG	R: ACAGAACCCAGGCCTTCTAC
ECI2	F: TCTCTGCGAAGCAAGGCAA	R: ATGGTGGCTGTAGTCATGTGC
NDRG1	F: CACAGCAACCCTGACCTGAT	R: GGTCTCGCCGACTGTTGTAA
CDK9	F: TAAGAACAGCCAGCCGAACC	R: ATCCGCAGAGCTGGCTAATG
FBXO25	F: GCACAGGTTCTCTGATGGCT	R: CAAGGTGTCTCCGTACTGCT
SLC25A25	F: AGATGTTGGGGAAGCCACAG	R: CCTTGTCTCCAGCCTTCACA
USP25	F: ATTTGGAGTTGGCTGTGGCT	R: AGTCTCCCTGAATGCCCTGT
